# An unusual case of twin anemia polycythemia sequence complicated by premature ductus arteriosus constriction of the recipient twin

**DOI:** 10.1515/crpm-2024-0027

**Published:** 2024-10-21

**Authors:** Ravi Chokshi, Kathryn McMullen, Shelly Soni, Robert Tunks, James O’Brien

**Affiliations:** Division of Maternal-Fetal Medicine, 12311 Penn State University Milton S. Hershey Medical Center, Penn State College of Medicine, Hershey, PA, USA; Department of Obstetrics & Gynecology, 12311 Penn State University Milton S. Hershey Medical Center, Penn State College of Medicine, Hershey, PA, USA; Richard D. Wood Jr. Center for Fetal Diagnosis and Treatment, Children’s Hospital of Philadelphia, Philadelphia, PA, USA; Division of Pediatric Cardiology, 12311 Penn State University Milton S. Hershey Medical Center, Penn State College of Medicine, Hershey, PA, USA

**Keywords:** monochorionic twins, twin anemia polycythemia sequence, fetal echocardiography, twin twin transfusion syndrome, premature ductal constriction

## Abstract

**Objectives:**

To add to the nascent literature on twin anemia polycythemia sequence by presenting a unique cardiac complication in the recipient twin.

**Case presentation:**

We describe a monochorionic diamniotic pregnancy complicated by twin anemia polycythemia sequence wherein the recipient twin developed signs of right heart failure secondary to premature ductus arteriosus constriction, requiring iatrogenic preterm delivery to avoid intrauterine demise.

**Conclusions:**

This case report introduces a previously undescribed complication of twin anemia polycythemia sequence and adds to the growing literature on this clinical entity.

## Introduction

The incidence of twin pregnancies continues to rise in the United States, with the use of assisted reproductive technology (ART) and increasing maternal age as notable contributing factors [1]. Monochorionic pregnancies, which account for around 20 % of twin pregnancies, have unique complications secondary to their shared placentation. This is attributed to the presence of vascular anastomoses in their placenta that allow for bidirectional blood flow. An imbalance in the net blood flow between the fetuses can lead to the development of complications such as twin-twin transfusion syndrome (TTTS), and twin anemia-polycythemia sequence (TAPS) [2].

In TTTS, the vascular anastomoses leading to unequal blood sharing are more pronounced causing significant fluid imbalance resulting in hypovolemia and oligohydramnios in the donor twin, and hypervolemia and polyhydramnios in the recipient twin [3]. TAPS in contrast is a slower, more subtle form of feto-fetal transfusion, that occurs through much smaller placental anastomoses and leads to anemia in the donor twin and polycythemia in the recipient twin [4]. While TTTS occurs in 10–15 % of monochorionic-diamniotic (MCDA) pregnancies, mandating surveillance, TAPS is a less common entity with a reported spontaneous incidence of around 3–5 % [4, 5]. The morbidity and mortality of TAPS primarily arises from the large inter-twin hemoglobin differences with subsequent hematological complications that can be severe in nature [6]. Tollenaar et al. [4] described 249 cases of spontaneous TAPS with a perinatal mortality rate of 15 %, a neonatal mortality rate of 4 % and serious neonatal morbidity rate of 33 %. More specifically, their findings noted worse outcomes for donor twins with a perinatal mortality of 22 % as compared to 7 % mortality rates for recipients. TAPS donor twins also had 4-fold higher odds of neurodevelopment impairment as compared to recipient twins. Their results underscore the severity of this disease process, and the need for further research into pregnancies complicated by TAPS.

We report here a case of MCDA pregnancy complicated by TAPS, wherein the recipient twin developed echocardiographic findings of premature ductal constriction and evidence of right heart failure necessitating delivery. A thorough literature search did not find prior reporting on this constellation of findings, making this a unique case to add to the growing knowledge on TAPS.

## Case presentation

Our patient was a 21yo G2P0010 diagnosed with spontaneous MCDA twins at 7 weeks gestation. Her obstetric history was relevant for a spontaneous early first trimester loss. Her past medical, surgical and family history were otherwise unremarkable. Her 12 week ultrasound with maternal fetal medicine (MFM) confirmed the MCDA nature of her twin pregnancy and she was counseled regarding associated prenatal complications including TTTS and TAPS. The patient underwent cell free DNA testing with results indicative of monozygotic male twins with a fetal fraction of 12.5 % and was low risk for common aneuploidies.

Given her monochorionic pregnancy, she started ultrasound surveillance for amniotic fluid discordance at 16 weeks gestation, followed by serial middle cerebral artery (MCA) peak systolic velocity (PSV) measurements for TAPS surveillance at 18 weeks as is the protocol at our institution. Her initial screening results were reassuring, until 21 w5d gestation when a discordance in amniotic fluid volume and MCA PSV was seen (Table 1). While not meeting strict Leiden criteria for TAPS [7, 8], she was referred to a regional fetal therapy center for consultation and further evaluation.

**Table 1:** Summary of US findings.

GA	DVP (twin A)	DVP (twin B)	Bladder (twin A)	Bladder (twin B)	UA S/D (twin A)	UA S/D (twin B)	MCA PSV MoM (twin A)	MCA PSV MoM (twin B)	EFW (twin A)	EFW (twin B)
21 w5d	3.6	8.04	Y	Y	5.6	5	1.32	<1		
21 w6d	2.8	6.9	Y	Y	3.21	3.03	1.4	0.71	443	442
22 w2d	2.5	7.8	Y	Y	3.49	3.18	0.84–1.08	0.72–0.86		
22 w5d	2.4	7.6	Y	Y	3.7	3.49	1.36–1.64	0.68–0.77		
23 w0d	3.8	9.2	Y	Y	3.09	3.17	1.29–1.34	0.56–0.63		
23 w2d	3.8	8.1	Y	Y	3.33	2.87	1.28–1.52	0.66–1.00		
24 w0d	2.8	7.6	Y	Y			1.52	1.03	596	593
24 w5d	2.5	9.4	Y	Y	3.01	3.06	1.11–1.18	0.67–73		
25 w1d	2.5	9.4	Y	Y	3.98	3.75	1.2–1.45	0.92–1.02		
25 w5d	2.8	6.8	Y	Y	3.2	2.46	1.3–1.37	1.02–1.04	760	768
26 w0d	2.6	6.6	Y	Y	3.09	2.79	1.08–1.43	0.73–0.92		
26 w1d	2.6	8.6	Y	Y	3.6	2.3	1.42	<1		

GA, gestational age; DVP, deepest vertical pocket, UA S/D umbilical artery systolic/diastolic, MCA PSV, MoM middle cerebral artery peak systolic velocity multiples of median, EFW, estimated fetal weight.

Evaluation at the fetal therapy center at 21 w6d noted discordant MCA dopplers, with 1.4 MoM for fetus A and 0.75 MoM for B nearing Leiden criteria for stage 1 TAPS. Close sonographic surveillance over the course of next few weeks noted progression to stage 2 TAPS along with discordant amniotic fluid but not meeting TTTS criteria [2]. Different management options were discussed including expectant management vs. laser photocoagulation for more definitive treatment, with the potential for early preterm delivery in either case. After extensive counseling, the patient opted for expectant management. At 24w5d, there was concern for evolving TTTS with worsening amniotic fluid discordance and cardiac dysfunction in the recipient. The option of laser photocoagulation was discussed again, and the couple continued to opt for expectant management. Patient was given a course of betamethasone in anticipation of preterm delivery. At 25 w5d, fetus B (polycythemic fetus) was noted to have moderate ductal constriction and increased combined cardiac output (CCO 746 mL/kg/min) on echocardiogram. Other cardiac changes included new onset tricuspid regurgitation (TR), mild dilation and hypertrophy of the right ventricle (RV) with concomitant mild decrease in RV function, and trivial pulmonary regurgitation (PR). Remainder of the ultrasound findings were stable. Follow-up echocardiogram in 48 h revealed persistent ductal constriction with decreasing combined cardiac output, worsening TR and worsening right heart function for fetus B. Given the deterioration of cardiac findings, antepartum admission for fetal monitoring was recommended.

**Figure 1:**
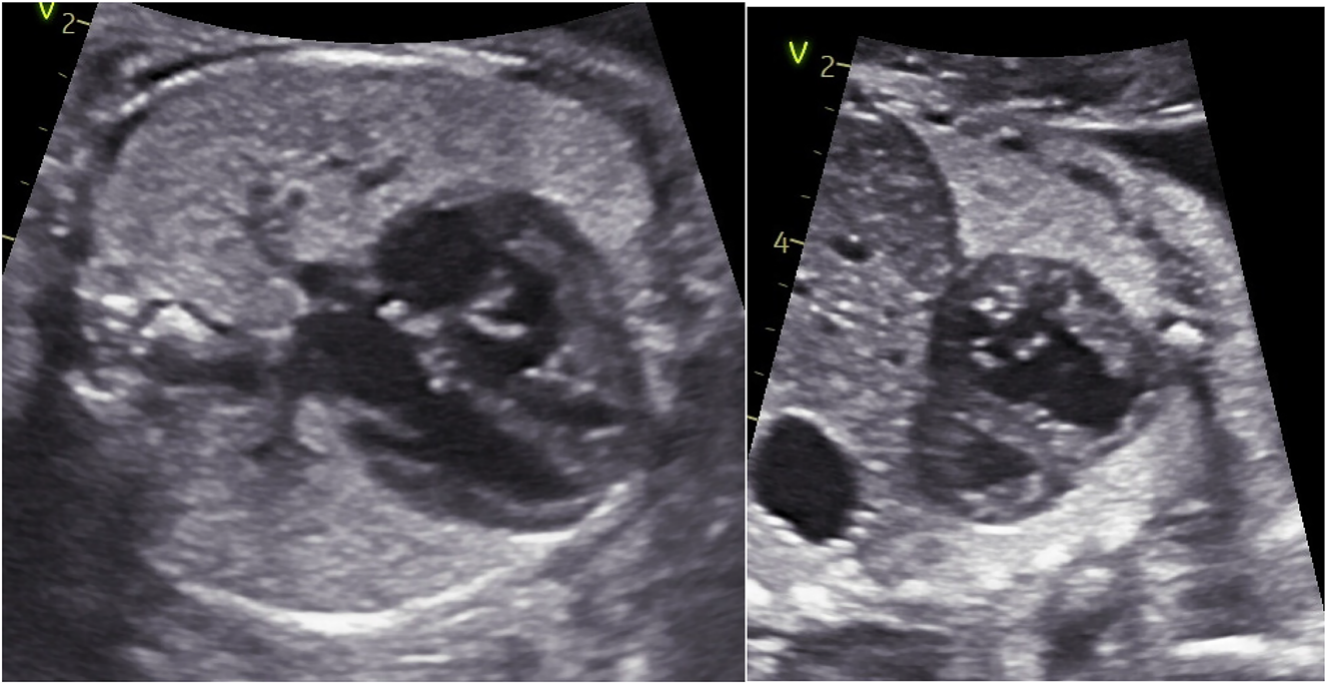
Fetus B with RVH and ventricular septum bowing.

**Figure 2:**
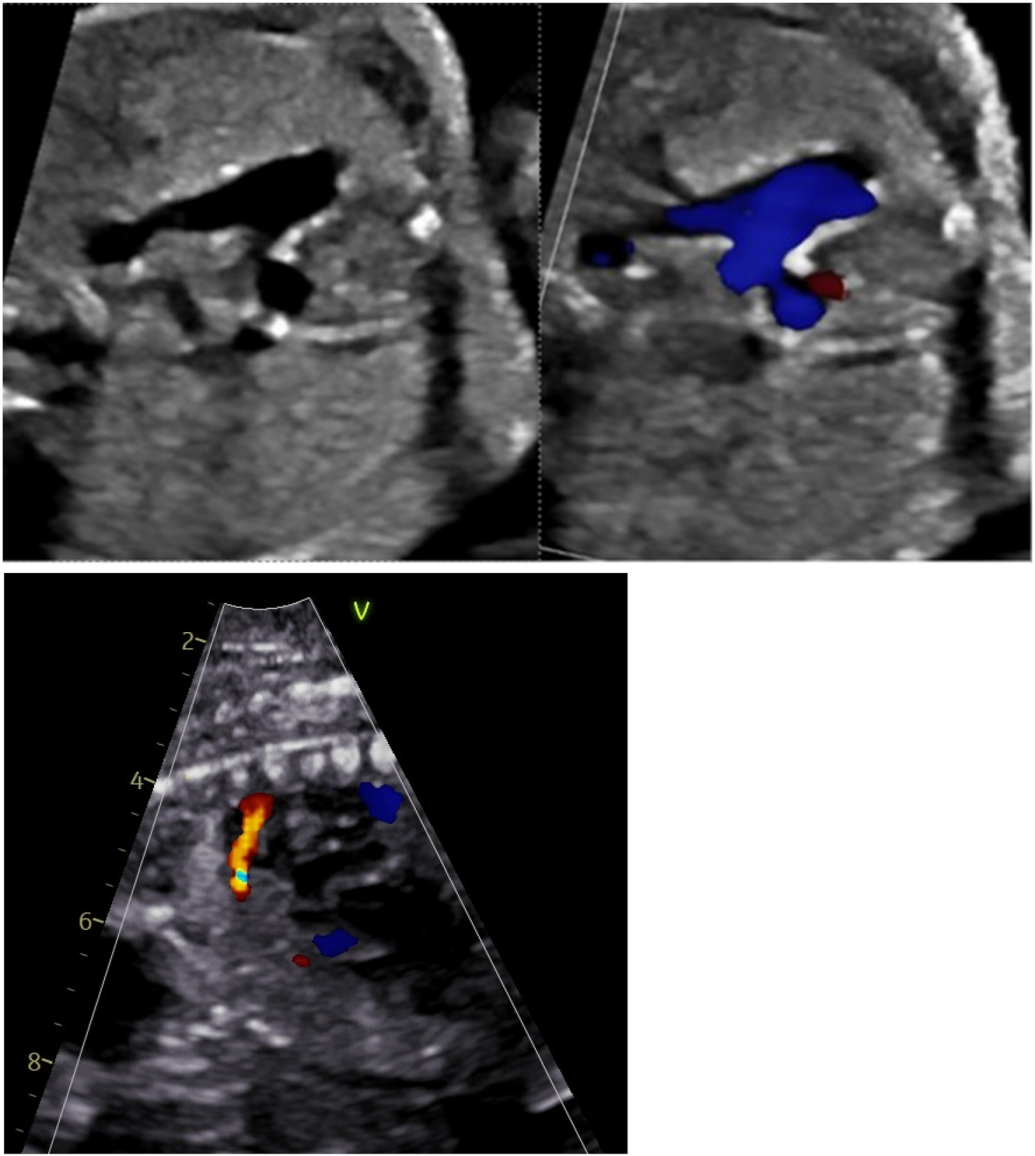
Fetus B with ductal constriction (transverse and sagittal view).

**Figure 3:**
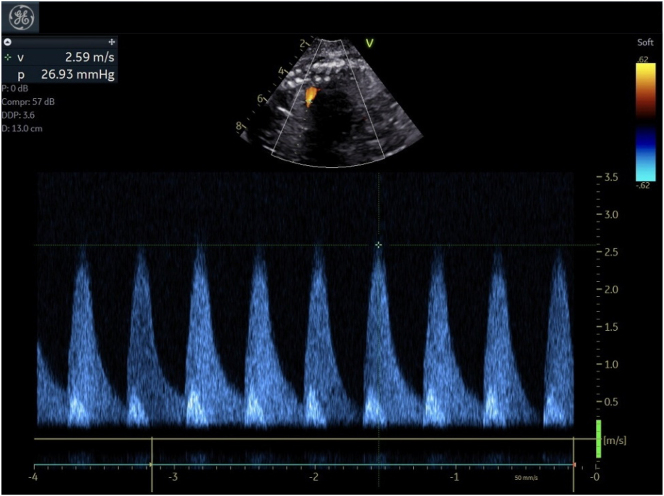
Fetus B-ductal arch velocity.

Patient was admitted to our tertiary care center and a rescue course of betamethasone was initiated at 26w0d. Fetal echocardiogram performed at 26 w1d demonstrated a moderately dilated and hypertrophied RV (Figure 1) with moderately depressed systolic function and mild TR for fetus B. Ductal arch constriction was noted by color Doppler and the ventricular septum bowed from right to left (Figures 1, 2 and 3) predicting RV pressure overload. No evidence of fetal arrhythmia was detected. Normal cardiac structure and function was confirmed for Fetus A.

Fetal monitoring via cardiotocography remained reassuring for both twins. At 26 w2d, fetal echocardiogram was repeated and demonstrated worsening findings for Fetus B, with the ductal arch appearing severely narrowed toward the pulmonary end. Peak velocity across the ductus was 2.7 m/s (PG: 27 mm Hg). The RV was severely dilated and moderately hypertrophied with moderately depressed systolic function. Worsening TR, with a peak gradient across the tricuspid valve of 50 mm Hg was detected, again consistent with RV pressure overload. There was no evidence of left ventricular outflow tract obstruction. Left ventricular systolic function remained preserved. No fetal arrhythmias were detected and there was no evidence of hydrops.

Due to worsening findings for twin B on fetal echocardiogram, after an extensive multidisciplinary discussion between MFM, Pediatric Cardiology and Neonatology and shared decision making with the couple, decision was made to proceed with delivery due to the high risk of progressive right heart failure and fetal demise for Twin B, with potential for secondary neurologic damage to Twin A. There was also concern regarding Twin B’s ductal constriction, and potential for neonatal pulmonary hypertension. Patient had completed rescue steroids, and a magnesium sulfate bolus and infusion were initiated for fetal neuroprotection. She underwent an uncomplicated low transverse cesarean delivery. Due to suspected TAPS, no delayed cord clamping was performed for either twin. Delivery details are in Table 2.

**Table 2:** Summary of delivery and neonatal findings.

Delivery:
1′LTCS at 26 w2d gestation Quantitative blood loss: 1,325 mL Due to concern for TAPS, no delayed cord clamping was performed for either twin.
Twin A, male:
– Birth weight 790 gms (32 % percentile)
– Required PPV in delivery room but good respiratory effort. Admitted to NICU on CPAP
– Apgar score – 6/8 (1 and 5 min)
– No initial cord gases obtained
– Hb 8.8 with Hct 25.6
– PLT 136
– Reticulocyte (%) 15.44
Twin B, male:
– Birth weight 800 gms (34 % percentile)
– Required PPV in delivery room, and was intubated at around 10 min of life due to poor respiratory effort.
– Apgar score – 5/7/8 (1, 5 and 10 min)
– 7.31/49/24.7/2.5 (v)/7.32/45/23.2/3.3 (a)
– Hb 20.3 with Hct 56
– PLT 216
– Reticulocyte (%) 8.42

1′LTCS, primary low transverse cesarean section; PPV, positive pressure ventilation; CPAP, continuous positive airway pressure; Hb, hemoglobin; Hct, hematocrit; PLT, platelet.

Twin A (donor twin) had a complicated postnatal course with profound intraventricular hemorrhage (IVH grade 3 and 4), respiratory failure secondary to pulmonary hemorrhages, persistent thrombocytopenia despite continuous blood product infusion, and sepsis. The hematocrit on admission was 26 % with a reticulocyte count of 15.4 %, confirming the postnatal diagnosis of TAPS with Twin A being the anemic/donor twin. Over NICU course, he had worsening bowel distension with imaging and blood cultures concerning for occult intestinal perforation. Due to high probability of not surviving surgery, an exploratory laparotomy was not performed. After extensive discussion the parents elected to transition to comfort care on day of life 8 with the neonate passing shortly thereafter.

Twin B (recipient twin) was initially intubated due to poor respiratory effort in the delivery room. He received surfactant and was extubated to non-invasive ventilation on day of life 1. His admission hematocrit was 56 % with a reticulocyte count of 8 %. Twin B’s initial postnatal echocardiogram demonstrated RV hypertrophy with qualitatively normal RV systolic function. RV pressures were estimated as systemic. A small patent DA was present with pressure restrictive, left-to-right shunting. Shunting across the patent foramen ovale (PFO) was bidirectional. The baby’s hemodynamic status remained stable and neither prostaglandin or pulmonary hypertension therapy were required. Serial imaging demonstrated progressive improvement in the baby’s right heart pressures. Twin B continues to receive care in the NICU progressing appropriately and meeting milestones. No evidence of cardiac complications, pulmonary hypertension or IVH has been identified.

## Discussion

To our knowledge, this case is the first to demonstrate a pregnancy with TAPS complicated by significant premature ductal constriction in the recipient twin, prompting early delivery secondary to right heart failure. This is an unanticipated consequence of TAPS, as typically the morbidity and mortality arises from the large inter-twin hemoglobin differences with subsequent hematological complications [6]. While cardiac dysfunction is seen in TTTS twins [9, 10], and this case could have a component of superimposed TTTS, ductal constriction is not a common finding for this syndrome adding to the unique nature of our case report.

The development of TAPS is an inherent risk of monochorionic placentation, occurring spontaneously in 3–5% of monochorionic twin pregnancies, and more commonly (2–16 %) after laser ablation for TTTS [7]. Lopriore et al. [11] first described and coined the term TAPS in 2007, underscoring the need for research in this nascent topic. While both TTTS and TAPS are secondary to shared placenta anastomoses, the types of anastomoses in TAPS differ from TTTS. TAPS seems to result from a few small arterio-venous (AV) anastomoses that allow for a chronic unbalanced intertwin transfusion with an indolent presentation. It is theorized that due to the slower more chronic nature of this twin-twin transfusion, there is adequate time for hemodynamic compensation between the twins and they do not develop the classic oligo-polyhydramnios as seen in TTTS [6]. Importantly, the derangements in the renin-angiotensin system (RAS) [12] seen in TTTS that lead to high renin levels in the donor twin and absent renin in the recipient twin, have not been reported in TAPS pregnancies [6], and may account for why cardiac dysfunction is not typically seen.

Our case noted premature constriction of the DA in the recipient twin. The DA is a crucial component of the fetal circulation, as it delivers most of the RV output to the descending aorta [13]. Premature constriction or closure of the DA would in effect lead to right ventricular outflow tract obstruction (RVOTO) with resulting RV failure. This is typically seen with maternal use of cyclooxygenase inhibitors such as nonsteroidal anti-inflammatory drugs (NSAIDs) [14]. Our patient reported no such use, and the physiologic mechanism of this finding in the recipient twin remains unclear. Literature review on TTTS notes the potential for RV dysfunction and failure, but not secondary to DA constriction. Lougheed et al. [9] noted that the recipient (hypervolemic) twin in TTTS was prone to biventricular hypertrophy, tricuspid regurgitation (TR) and biventricular dysfunction. They reported a 9.6 % incidence of RVOTO in the recipient twin, with pulmonary stenosis or atresia factoring in most cases. *In utero* ductal constriction was not seen in their study. Pruetz [15] described a ‘circular shunt physiology’ in recipient twins, with a significant number of these fetuses developing RVOTO in the post-natal period. No reported cases of premature ductal closure or stenosis were noted^13^. Neonatal patent ductus arteriosus requiring treatment was defined as a severe neonatal morbidity and was a primary outcome of a study by Tollenaar et al., but *in utero* ductal constriction was not specifically reported as a complication^13^. While our patient never met strict criteria for TTTS, there was known amniotic fluid discordance indicating that TTTS and TAPS likely operate on a spectrum with the potential for shared complications, with a recent publication noting that TAPS co-exists in 15 % of TTTS cases [16].

In summary, we report on an interesting case of an MCDA pregnancy complicated by antenatal diagnosis of TAPS, wherein the recipient twin developed echocardiographic findings of premature ductal constriction and evidence of right heart failure necessitating delivery. Despite aggressive care, the outcome for Twin A (donor) was poor, which was anticipated by the literature [4]. We were unable to find this constellation of findings previously reported, adding value to our current knowledge on TAPS.
